# Supplementary material for: Comparison of Nicotine Dependence and Biomarker Levels among Traditional Cigarette, Heat-Not-Burn Cigarette, and Liquid E-Cigarette Users: Results from the Think Study
Source: Int J Environ Res Public Health. 2021 Apr 29;18(9):4777. doi: 10.3390/ijerph18094777 (PMC8124521; doi:10.3390/ijerph18094777)
Supplement: Supplementary file 1 [file ijerph-18-04777-s001.zip › ijerph-1161240-supplementary.pdf]

Supplementary Information

Comparison of Nicotine Dependence and Biomarker Levels among Traditional Cigarette, Heat-Not-Burn Cigarette, and Liquid E-Cigarette Users: Results from the Think Study

Guillaume Rudasingwa [1], Yeonjin Kim [2], Cheolmin Lee [3], Jeomkyu Lee [4], Seunghyun Kim [4] and Sungroul Kim [1,2,*]

[1] Integrated Research Center of Risk Assessment, Soonchunhyang University, Soonchunhyang-Ro 22, Asan 31538, Korea; guillaumer1992@gmail.com

[2] Department of ICT Environmental Health System, Graduate School, Soonchunhyang University, Soonchunhyang-Ro 22, Asan 31538, Korea; duswls0911@naver.com

[3] Department of Family Medicine, Healthcare System Gangnam Center, Seoul National University Hospital, Seoul 06236, Korea; bigbangx@snuh.org

[4] Division of Respiratory and Allergy Disease Research, Department of Chronic Disease Convergence Research, National Institute of Health (NIH), Korea Disease Control and Prevention Agency (KDCA), Osong 28159, Korea; nihdot@korea.kr (J.L.); shkims00@korea.kr (S.K)

* Correspondence: Sungroul.kim@gmail.com; Tel.: +82-41-530-1266

Supplementary Table 1. Demographic and socioeconomic status used for evaluation of the association of urinary cotinine with smoking amount adjusted for nicotine addiction levels.

		Nonsmokers (n=63)			Cigarette only (n=403)			Liquid e-Cig only (n=24)			HNB e-Cig only (n= 76)		
		N	%	p-value	N	%	p-value	N	%	p-value	N	%	p-value
Gender	Female	39	61.9	<0.001	46	11.4	<.0001	1	4.2	<.0001	5	6.6	<.0001
	Male	24	38.1		357	88.6		23	95.8		71	93.4	
Age group (Years)	~ 29	18	28.6	<0.001	149	37.2	0.1853	7	29.2	0.232	15	20.0	<.0001
	30 ~ 49	41	65.1		133	33.2		12	50.0		43	57.3	
	50 or older	4	6.3		119	29.7		5	21.8		17	22.7	
Education	Univ	52	82.5	<0.001	276	68.5	<.0001	22	91.7	<.0001	71	93.4	<.0001
	High school	11	17.5		127	31.5		2	8.3		5	6.6	
House income (M, Won)	5 +	46	73.0	<0.001	217	53.9	0.123	17	72.5	0.004	59	77.6	<.0001
	4.99 or less	17	27.0		186	46.2		7	27.5		17	22.4	
Marrage status	Married	39	61.9	<0.001	193	47.9	0.397	13	54.2	0.527	54	71.1	0.0002
	Others	24	38.1		210	52.1		11	45.8		22	29.0	
Fagerstrom score	1	NA	NA	NA	157	38.7	<.0001	17	70.8	0.017	43	56.6	<.0001
	2				117	29.2		6	25.0		25	32.9	
	3				110	27.4		1	4.2		8	10.5	
	4				19	4.7							
Cigarette per day	~ 5	NA	NA	NA	55	13.7	<.0001	9	37.5	0.0312	6	8.0	<.0001
	6 ~ 10				113	28.2		5	20.8		31	41.3	
	11 ~ 15				86	21.4		4	16.7		22	29.3	
	16 ~ 20				111	27.7		2	8.3		13	17.3	
	21 ~ 25				11	2.7		.			3	4.0	
	26 ~ 30				20	5.0		.			.		
	31 +				5	1.3		4	16.7		.		

* Sum of each cell in each variable may not equal to total due to missing value

** p-value from Chi-square test

Supplementary Table 2. Associations of biomarker concentration levels with smoking amount adjusted for nicotine addiction levels among single-type cigarette users (TC, EC, or HNB).

	Cigarette (R^2 = 0.12)		Liquid e-Cig (R^2 = 0.49)		HNB e-Cig (R^2 = 0.27)	
	beta	p-value	B	p-value	B	p-value
Time to first cigarette (Ref: 61+min)						
~5	**0.582**	***0.001***	0.647	*0.680*	-0.060	*0.895*
6~30	**0.287**	***0.040***	3.647	*0.223*	-0.267	*0.362*
31~60	0		0	.	0	.
Difficulty to not smoke (Ref: No)						
Yes	-0.222	*0.126*	4.333	*0.009*	-0.024	*0.945*
Type of cigarette most like (Ref: others)						
Morning	0.071	*0.583*	0.030	*0.992*	-0.248	*0.463*
More smoking in morning (Ref: No)						
Yes	-0.051	*0.721*	-2.767	*0.233*	0.305	*0.385*
Smoking when sick (Ref: No)						
Yes	0.061	*0.626*	-2.398	*0.380*	**1.037**	***0.001***
Cigarette per day (Ref: ~5 cigarettes)						
6 ~ 10	0.230	*0.299*	0.109	*0.939*	**1.712**	***0.007***
11 ~ 15	**0.714**	***0.002***	2.503	*0.176*	**1.917**	***0.004***
16 ~ 20	**0.569**	***0.015***	-1.692	*0.657*	**2.180**	***0.002***
21 ~ 25	0.640	*0.088*		.	**2.002**	***0.038***
26 ~ 30	**0.798**	***0.015***		.	0.000	.
31 +	0.466	*0.351*	**2.028**	***0.005***	0.000	.
Gender (Ref: Female)						
Male	0.287	*0.138*	NA	*NA*	-0.159	*0.772*
Age group (Ref:50 + years)						
~29	0.224	*0.117*	-1.830	*0.067*	-0.052	*0.901*
30~49	0.234	*0.095*	-0.671	*0.600*	-0.027	*0.928*
House income (Ref: 5M Won)						
4.99 or less	-0.023	*0.839*	-1.232	*0.343*	0.387	*0.262*

* Sum of each cell in each variable may not equal to total due to missing values
